# Barriers to Telemedicine Use: Qualitative Analysis of Provider Perspectives During the COVID-19 Pandemic

**DOI:** 10.2196/39249

**Published:** 2023-06-26

**Authors:** Milan Patel, Hanna Berlin, Abishek Rajkumar, Sarah L Krein, Rebecca Miller, Jessie DeVito, Jake Roy, Margaret Punch, Chad Ellimootti, Alex F Peahl

**Affiliations:** 1 Department of Urology Loyola University Medical Center Maywood, IL United States; 2 University of Michigan Medical School Ann Arbor, MI United States; 3 Center for Clinical Management Research VA Ann Arbor Healthcare System Ann Arbor, MI United States; 4 Institute for Healthcare Policy and Innovation University of Michigan Ann Arbor, MI United States; 5 Virtual Health Michigan Medicine Ann Arbor, MI United States; 6 Department of Obstetrics and Gynecology University of Michigan Ann Arbor, MI United States; 7 Department of Urology University of Michigan Ann Arbor, MI United States

**Keywords:** telehealth, virtual visits, public health crisis, barriers and facilitators, provider perspectives, implementation, access, telehealth, health care, patient care

## Abstract

**Background:**

Though telemedicine is a promising approach for removing barriers to care and improving access for patients, telemedicine use for many medical specialties has decreased from its peak during the acute COVID-19 public health crisis. Understanding the barriers and facilitators to the maintenance of web-based visits—one key component of telemedicine—is critical for ensuring the continuous availability of this service for patients.

**Objective:**

The purpose of this study is to describe medical providers’ perceived barriers and facilitators to the continued use of web-based visits to inform quality improvement efforts and promote sustainability.

**Methods:**

We performed a qualitative content analysis of free-text responses from a survey of medical providers administered from February 5-14, 2021, at a large, midwestern academic institution, including all providers from medical professions that offered telemedicine (eg, physicians, residents or fellows, nurse practitioners, physicians assistants, or nurses) who completed at least 1 web-based visit from March 20, 2020, to February 14, 2021. The primary outcome was the experience of providing web-based visits, including barriers and facilitators to continued usage of web-based visits. Survey questions included 3 major domains: quality of care, technology, and satisfaction. Responses were coded using qualitative content analysis and further analyzed through a matrix analysis to understand the providers’ perspectives and elucidate key barriers and facilitators of web-based visit usage.

**Results:**

Of 2692 eligible providers, 1040 (38.6%) completed the survey, of whom 702 were providers from medical professions that offered telemedicine. These providers spanned 7 health care professions and 47 clinical departments. The most common professions represented were physicians (486/702, 46.7%), residents or fellows (85/702, 8.2%), and nurse practitioners (81/702, 7.8%), while the most common clinical departments were internal medicine (69/702, 6.6%), psychiatry (69/702, 6.6%), and physical medicine and rehabilitation (67/702, 6.4%). The following 4 overarching categories of provider experience with web-based visits emerged: quality of care, patient rapport, visit flow, and equity. Though many providers saw web-based visits as a tool for improving care access, quality, and equity, others shared how appropriate selection of web-based visits, support (eg, patient training, home devices, and broadband access), and institutional and nationwide optimization (eg, relaxation of licensing requirements across state borders and reimbursement for phone-only modalities) were needed to sustain web-based visits.

**Conclusions:**

Our findings demonstrate key barriers to the maintenance of telemedicine services following the acute public health crisis. These findings can help prioritize the most impactful methods of sustaining and expanding telemedicine availability for patients who prefer this method of care delivery.

## Introduction

The COVID-19 pandemic has triggered remarkable growth in telemedicine. Telemedicine services such as web-based visits offered health care delivery alternatives that limited viral exposure and the use of resources while maintaining necessary medical services. Health care workers across professions and specialties rapidly adopted telemedicine to provide diverse services for their patients—from prenatal visits to preoperative consults to psychiatric counseling [1-3]. As a result, web-based visits increased from <1% of all outpatient encounters prior to the public health crisis to at least 30% in the initial months of the pandemic [4-7].

Telemedicine is a promising avenue for improving patients’ health care convenience and access by reducing care barriers like travel and childcare needs [8-12]. Yet, following its rapid uptake in the acute public health crisis, telemedicine usage has declined for some specialties, while others, like psychiatry, have maintained high levels of usage [6,7,13]. Certainly, some decline in telemedicine services was expected with the relaxation of social distancing and decreased risk of viral exposure. Specialties that do not require regular physical examinations or laboratory data may be more conducive to telemedicine; however, there are at least some applications for telemedicine in all specialties, from incision checks to medication adjustments. The uneven decline in the use of telemedicine suggests that other factors may contribute to whether practices maintain even modest levels of telemedicine offerings. The challenges at the patient, provider, and institution levels may preclude the maintenance of telemedicine services following the acute public health crisis. Specifically, concerns about care quality, supporting technology, and equity have been highlighted as the potential roadblocks to the continued widespread availability of telemedicine services [1,14,15]. Yet to date, the barriers and facilitators of sustained telemedicine usage are insufficiently described.

Health care professionals who deliver telemedicine services are uniquely positioned to understand the multilevel barriers, facilitators, and solutions needed to support their continued use. Our institution rapidly scaled web-based visits during the acute COVID-19 pandemic, increasing from 22 visits per day in February 2020 to a peak of 1823 per day in December 2021, with a subsequent decline. Thus, we conducted a mixed methods survey of providers to understand the drivers of telemedicine maintenance and inform quality improvement efforts necessary to support continued telemedicine services.

## Methods

### Ethical Considerations

This study was a qualitative content analysis of the free-text responses collected from a provider survey administered during February 5-14, 2021, to providers at Michigan Medicine who had completed at least 1 web-based visit. The University of Michigan Institutional Review Board deemed this survey project unregulated.

### Overview

In response to the COVID-19 public health crisis, in March 2020, our institution rapidly scaled web-based visit capability for all providers (eg, physicians, nutritionists, and social workers). The providers were encouraged to use the recommended electronic health record–based platform; however, in concordance with emergency Health Insurance Portability and Accountability Act (HIPAA) regulations, other communication technologies (eg, Zoom) were deemed acceptable. A web-based help desk was available for technological issues. Individual specialties varied in web-based visit implementation, including (1) provider location (home or in office), (2) visit scheduling (web-based–only blocks, interspersed with in-person visits), (3) patient rooming (medical assistant [MA]–reviewed patient information, no MA check-in), and (4) providers’ use of clinic or personal devices.

This survey was developed with the Virtual Care Team at our institution for quality improvement. Questions addressed the key domains previously identified as the potential drivers of telemedicine maintenance, including the following:

1. Quality: providers’ ability to deliver medical services and develop rapport with patients
2. Technology: the degree to which patients and providers were able to use and complete video visits;
3. Satisfaction: providers’ overall experience with video visits and willingness to continue them following the acute public health crisis, including burnout and payment
4. Equity: the effect of telemedicine on existing health care inequities

Questions were asked in multiple-choice format, with free-text responses available. All questions were reviewed by an expert in telemedicine (CE) and survey methodology (AP). The survey was pilot-tested and approved by local telemedicine champions, with no recommended revisions, prior to deployment. The final survey included 7 multiple-choice questions and 2 free-text response questions. Participants were also able to provide free-text responses to give further context to their multiple-choice selections (Multimedia Appendix 1). All qualitative responses were included in the content analysis. The survey was administered through the web-based Qualtrics platform.

We performed a qualitative content analysis of the free-text responses. Qualitative data were uploaded to MAXQDA software (version 20.4.0; VERBI GmbH) for management and analysis. Three authors (AP, MP, and HB) immersed themselves in the data and generated a preliminary codebook using inductive reasoning to construct initial codes using the constant comparison method [16-18]. This codebook was applied to the first 50 free-text responses for each question. The 3 authors then met to discuss the codebook, resolve discrepancies, and develop definitions and examples for each code. Following this initial coding consensus meeting, code definitions were revised and 3 codes were added to the codebook, for a total of 63 codes. Two authors (MP and HB) jointly coded groups of 20 responses until reaching 100% agreement, and then coded the remaining responses independently. The authors met frequently, and a third author (AP) resolved the coding discrepancies if necessary.

The final codebook included 2 general groups of codes: codes describing individuals’ experiences with web-based visits and codes describing their ideal future state. For codes describing actual experiences, we used a matrix analysis technique to further understand the providers’ perspectives, comparing barriers and facilitators within each code in a grid. A comparison of these responses allowed us to identify the most salient drivers of telemedicine maintenance. The codes related to the providers’ ideal future state were presented separately as potential recommendations for improving telemedicine delivery.

## Results

### Overview

Of the 2692 providers at Michigan Medicine who had completed at least 1 web-based visit, 1040 (38.6%) completed the survey, including 702 providers from medical professions that offered telemedicine. These providers represented 4 health care professions, including physicians (486/702, 46.7%), residents or fellows (85/702, 8.2%), and nurse practitioners (81//702, 7.8%) from 47 clinical departments, including internal medicine (69/702, 6.6%), psychiatry (69/702, 6.6%), and physical medicine and rehabilitation (67/702, 6.4%; Table 1).

**Figure 1:**
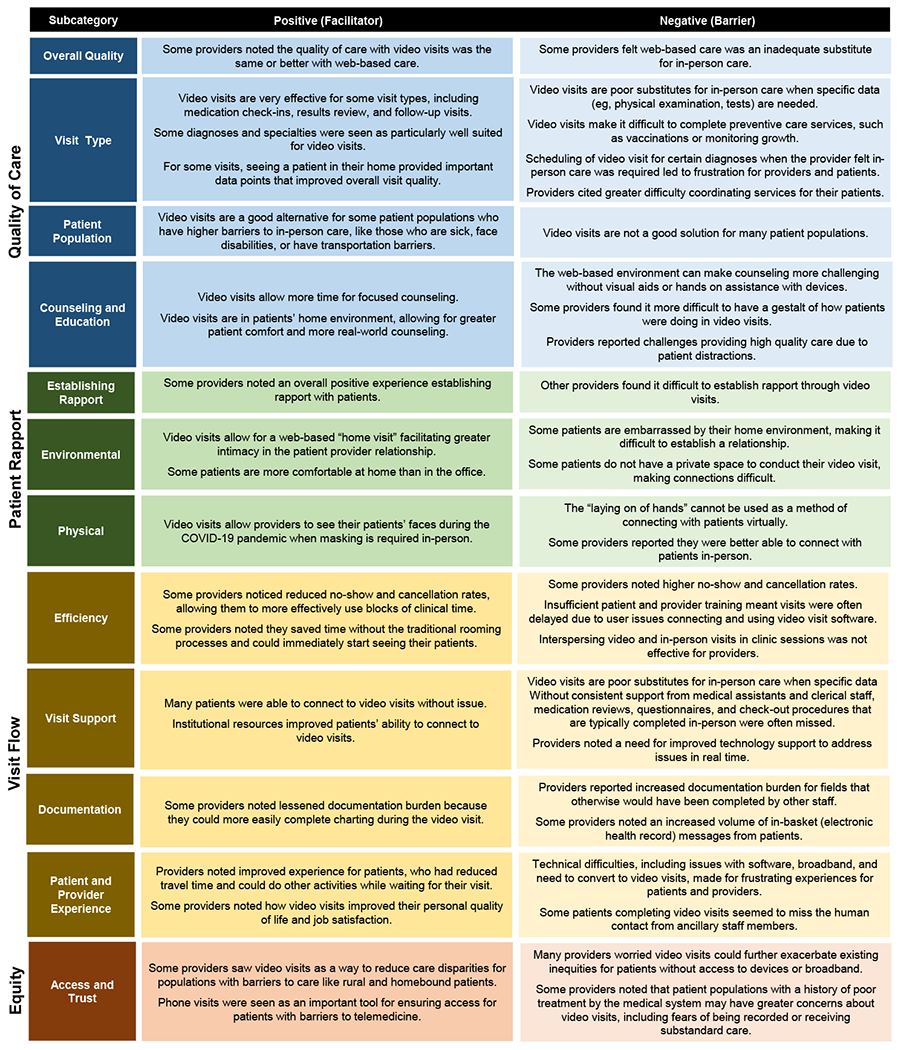
Matrix coding summary of provider telemedicine experience.

The following 4 overarching categories of provider experience emerged: quality of care, patient rapport, visit flow, and equity. See Figure 1 for key domains and Multimedia Appendix 2 for full quotations by domain.

**Table 1:** Professions and top clinical departments of survey participants.

Characteristics		Participants, n (%)
**Professions**		
	Physician	486 (46.7)
	Resident or fellow	85 (8.2)
	Nurse practitioner	81 (7.8)
	Physician assistant	50 (4.8)
**Top clinical departments**		
	Internal medicine	69 (6.6)
	Psychiatry	69 (6.6)
	Physical medicine and rehabilitation	67 (6.4)
	Neurology	51 (4.9)
	Obstetrics and gynecology	50 (4.8)
	Family medicine	39 (3.8)
	General medicine	39 (3.8)
	Hematology and oncology	38 (3.7)
	Gastroenterology and hepatology	37 (3.6)

### Quality of Care

Providers’ perceived ability to deliver high-quality medical care through telemedicine was mixed. While some believed “the quality of care is also same, if not better, than in person,” others found video visits were an inadequate substitution for in-person care, reporting, “the quality of video visits is garbage.” Perceptions of quality were driven by 4 key factors: appropriate selection of visit modality, availability of clinical services, patient population, and effects of web-based visits on communication.

For appropriately selected specialties (eg, psychiatry), visit types (eg, medication check-ins), and diagnoses (eg, chronic disease management), web-based visits could have the equal quality to in-person care. In some cases, appropriately selected web-based visits even improved patients’ care—seeing patients in their home environment provides a better examination than we get in clinics. This web-based “home visit” provided richer data than an office examination. Other times, web-based visits were an inadequate substitute for in-person care, such as for new patients or when specific laboratory data were needed. Scheduling web-based visits when in-person care was more appropriate contributed to low-quality web-based visits for some patients: “... when it became clear a physical exam was necessary.” More appropriate triaging of visits was seen as critical for “much more efficient experience[s].”

Providers highlighted physical examination maneuvers, tests, and additional services that could not be delivered digitally, including measuring limb strength, listening to fetal heart tones, and electrocardiograms. Web-based physical examinations had several shortcomings, including poor lighting, image quality, and camera angles. Similarly, providers found it difficult to get a “gestalt” of how a patient was doing through body language and nonverbal cues. Finally, providers noted coordination of services (eg, vaccines and referrals) was more complex with web-based visits: “I can’t walk down the hall and ask the social worker if she can see the pt while she is in clinic.” Not being able to “walk down the hall” meant providers needed to spend more time “on the back end” arranging care for their patients.

Web-based visits were seen as more appropriate for some patient groups than others. For example, patients who were sick, with physical disabilities, were concerned about viral exposure or had out-of-town family members greatly benefited from web-based visits. In contrast, providers worried that other patients, including children and people with a history of hearing loss or trauma, might be disadvantaged by this modality.

Some providers noted improved communication in web-based visits, saying they allowed “more time to address their [patients’] issues.” Others noted how web-based tools, including screen sharing, facilitated better patient counseling. One provider commented on how web-based counseling translated into improvements in health behaviors: “I have been successful in counseling, and when I see the patients back in office, am pleasantly surprised with improvements reported by changes made with behavioral modifications discussed during initial video visit.”

Not all providers experienced improvements in patient counseling. Some noted how the web-based environment made teaching more difficult as visual aids and hands-on assistance with devices (eg, inhalers and insulin pumps) were less accessible. Additionally, nonclinical distractions, including driving, family members, or a lack of privacy, made conversations more difficult between patients and providers, leading providers to “worry that pieces of information may be getting lost.” These patients could not fully engage in web-based conversations.

### Patient Rapport

Web-based visits both improved and challenged providers’ ability to connect with their patients. Some noted equal or even improved patient rapport through web-based visits. These positive experiences were driven by the ability to see patients in their home environment, which “creates a new kind of intimacy, kind of like a modern-day house call.” The web-based “house call” provided new depth to the patient-provider relationship beyond the sterile clinical setting. Other providers noted patients seemed more at ease in their own homes “rather than being in an office setting as a ‘patient.’” Being able to see patients’ faces and better interpret their facial expressions was also advantageous during the pandemic mask mandates.

In contrast, some providers found it challenging to connect in the web-based environment, reflecting that “this technology is eroding the doctor-patient relationship significantly.” Providers identified 3 sources of this challenge: lack of privacy, invasiveness of web-based visits, and the inability to connect through touch. Providers reported that patients sometimes could not find a private space for their visit or were multitasking, making it particularly difficult to have a safe space to build a patient-provider relationship. Similarly, the invasion of privacy in web-based visits was intrusive for some patients: “Many people struggle with clutter and are embarrassed to have people in the home.” Thus, for some patients, web-based visits represented a loss of the “neutral space” and subsequent comfort provided by clinical settings.

Providers also commented on the loss of “laying on of hands,” which they saw as an important part of the therapeutic alliance. This was particularly true for providers in specialties where the physical examination was central to the visit: “I am in a specialty that requires more hands-on physical exam. That helps build trust and rapport that is impossible over video.” As a result, building equal trust and rapport in a web-based environment felt impossible for some providers.

### Visit Flow

Providers reported that web-based visits improved efficiency through better patient show rates, eliminating patient care delays, and allowing for real-time documentation. The ability to easily connect to technology was crucial for realizing these gains. Additionally, internal resources, including MA rooming assistance for some visits or a medical student initiative that provides support to geriatric patients connecting to web-based services, were considered helpful for ensuring patients could complete web-based visits.

Some providers noted reduced appointment cancelations and patient no-shows for their web-based visits compared to in-person care, particularly for patients with barriers to care like low-income patients, those with disabilities, and those who lived far from the clinic. Web-based visits also alleviated other common reasons for missed appointments, like weather or traffic delays.

Providers noted other advantages to appointment efficiency, including time saved rooming patients: “There is less transition time, and I'm spending more time with patients as opposed to going from point A to point B.” Less “transition time” left providers with more time with their patients. For some providers, web-based visits also facilitated more efficient documentation, as they could maintain eye contact while typing. Improved efficiency contributed to higher satisfaction for both providers *and* patients. Providers noted how reduced travel time and coordination led to a better patient experience.

If it is a counseling or other appointment that an exam is not required, I would argue that the care is exactly the same if not better as patient's don't have the added stress of travel/parking/checking in/out.

The combination of improved efficiency and higher patient satisfaction led to improved job satisfaction for many providers: “This leads to substantially higher satisfaction for patients and myself... which improves my home life as well.” In sum, for many providers, web-based visits offered a pathway to more efficient, streamlined patient care, resulting in a better patient and provider experience.

Some providers experienced frustrating challenges with web-based care, including higher no-show rates, technical difficulties, and insufficient support—all resulting in lower satisfaction. The providers shared a variety of possible reasons for higher no-show rates for web-based visits, including administrative errors, technical difficulties, or patients’ perceived differences between canceling in-person and web-based visits.

Additionally, providers perceived delays in care due to insufficient patient and provider training on web-based platforms and also expressed issues accessing the electronic health record app, using the software, and even turning on video and sound. These burdens were felt even more when web-based visits were interspersed with in-person care. Similarly, providers described a need for better real-time technical support for patients “when they are struggling” at the beginning of a visit. One provider suggested the use of a “tech-barrier interpreter,” similar to interpreters used for patients with language barriers.

Web-based visits were made even more “hectic” by the lack of support some providers perceived in reviewing the patient’s history and medications, completing questionnaires, and gathering historical data. Though these processes were completed through an automated e-check-in, providers perceived this information as less reliable than in-person data entered by MAs, clerks, and trainees, who provided at-the-elbow assistance for in-person care.

Other provider frustrations with clinical workflow included more onerous documentation, technical difficulties, and an increased volume of postvisit follow-up work. Some providers noted challenges in documenting in real time through web-based visits and reported more unresolved postvisit documentation. Providers noted issues with inconsistent internet connections and the need to convert to telephone encounters from video visits, resulting in “frequent technical hurdles which are frustrating to patients and providers.” As a result, some providers saw web-based visits as inefficient and disruptive.

### Equity

Providers saw web-based visits as both a facilitator and a barrier to equity. Providers highlighted how web-based visits improved access for specific populations, including patients with disabilities, rural patients, and patients with poor access to care. Still, some providers worried that web-based visits might further exacerbate existing inequities for patients without access to devices or broadband or who did not speak English. As a result, some providers worried that the push for web-based care would result in some patients being left behind. As one provider reflected, “I do worry a lot about my patients who have limited technology access.”

Many providers emphasized the need to maintain video and phone visit options to ensure access for all patients, noting specific populations that might benefit from phone visits, including older adults, patients with disabilities or low technology literacy, and patients without access to needed technology (eg, those in rural areas).

Finally, providers shared that some populations, particularly those who have faced historical injustices, expressed concerns about privacy. As one described, “there is also some mistrust of VV [virtual visits] in our at-risk populations. I have had some tell me they don't want the VV option, just phone, as they are concerned about being recorded.” This fear of inadequate privacy led some patients who may have benefited from web-based visits to decline the service. Rather than improving convenience and access, web-based visits “erod[ed] the doctor-patient relationship significantly,” increasing existing inequities.

### Ideal Future State

Providers noted several institutional and national changes needed to realize the ideal future state of telemedicine delivery (Table 2). Institutionally, providers emphasized the importance of providing patients with tools to ensure web-based visits were of the highest quality, including a loaner program for tablets, having home visiting nurses complete vital signs and components of the physical examination, and helping patients obtain broadband access. Some providers perceived that institutional targets for web-based visits challenged providers’ autonomy to determine visit type, limited the patient-centeredness of care, and at worst, reduced patients’ access to appropriate visit types: “Having arbitrary goals for the number of virtual visits is insulting. It suggests that we should practice to achieve a metric.” From the provider’s perspective, incentivizing “arbitrary goals” for web-based care limited their ability to use shared decision-making to decide on appropriate web-based visit use.

On the state and national levels, providers identified 3 key policy recommendations: (1) removal of state-based licensing restrictions, (2) maintenance of parity for video- and audio-only visits, and (3) support for broadband expansion and access. Providers noted how state-based licensing prevented patients who were “traveling the farthest” from taking advantage of web-based visits. This limited the continuity of care for the patients who traveled during the winter or lived over state lines. As emphasized above, parity between video- and audio-only visits was a top priority for maintaining equity in new models. Finally, providers shared ideas for partnering with lawmakers and communities to expand broadband access. While some highlighted the need to “invest in universal access to broadband internet access, just like water and electricity and roads,” others envisioned building infrastructure in community physicians’ offices or libraries. In sum, providers envisioned a future where policy supports equitable access to high-quality web-based visits for all patients who prefer this modality, across state borders, and through both video- and audio-only platforms.

**Table 2:** Specific policy concerns.

Domain	Concern	Ideal future state reflected by providers
No out-of-state limits	Providers noted how licensing barriers prevented accessibility for out-of-state patients.	*Difficult for hospital with large catchment area like U of M to not be able to offer video visits for people traveling the farthest.* *The other group is patients who live out of state—enabling providers to see these patients would improve access to and continuity of care (for example when patients go to Florida for the winter, or those who live in Toledo).*
Parity of audio or video	Maintaining equity for video- and audio-only visits was seen as a critical issue for ensuring access and equity.	*Lobby for phone reimbursement equivalent to other forms of care, since telephone is often more accessible than video connection.* *The obsession with video visits definitely is a DEI issue. The more disadvantaged of our patients are the ones less likely to be able to do video visits and I am shocked the University has not been more aware of this. A good way to help this would be to endorse phone visits. Everyone has and knows how to use a telephone. There are seldom technical issues when calling someone on a phone. Stop disparaging phone visits.*
Need for public partnership for broadband	Many providers saw policy changes and partnerships with community institutions as promising avenues for expanding access to video visits.	*Encourage our legislature to invest in universal access to broadband internet access, just like water and electricity and roads. The internet is a necessary utility and not a luxury, and pandemic should have erased any doubt about this.* *For rural communities that have poor or no internet, would it be worth providing pts with a list of sites where they could find a private room with internet (e.g. library)? Similarly, for low income communities (or maybe ALL communities), would it be worth providing a list of sites where they could find a private room with computer & internet (e.g. library)?*
Need for patient education	Providers identified a need for classes to help patients with technology skills.	*Would be nice to offer a virtual video visit support (101) class for those who want to become more tech savvy.* *If we could continue to optimize patient education resources or real-time assistance to help them with establishing the video connections, that would be great, and then it would be 100% of the way there. I'd say it's 80%-90% of the way there right now. :)*
Need for devices	Providers saw devices as crucial for improving patients’ access to video visits.	*Providing patients needing frequent video visits with devices (loaners). Facilitating internet access for families.* *Consider pushing out technology (low-cost tablets with 4G or 5G capability) to our patients in high poverty areas.*
Flexible institutional policy	Providers noted frustration with several institutional video visit policies, including establishing a required proportion of video visits and removing needed social support services.	*Michigan Medicine has also unfortunately reduced prioritization of social work support in the health system and have cut staff who previously had the time to take the extra time to work with patients and families who had less access and lower resources but now they are required to move too quickly through their work in scheduling to even learn of people's needs. It costs money to serve underserved populations and unless the institution backs this priority with financial resources, this will be words and not action.* *I strongly feel that the number of virtual visits should be determined by shared physician or provider and patient decision-making. Having arbitrary goals for the number of virtual visits is insulting. It suggests that we should practice to achieve a metric rather than practice in a way that is medically reasonable and personally acceptable to patients.*

## Discussion

### Principal Results

In this cross-sectional survey, providers across multiple specialties and professions shared the benefits and challenges of providing telemedicine in 4 key domains: quality, patient rapport, visit flow, and equity. These nuanced experiences highlight how telemedicine may help address many pressing issues in health care but is not a stand-alone solution. As we move further away from the COVID-19 public health crisis, how to best integrate telemedicine, and to what extent and for which specialties, remains unknown [19,20].

### Comparisons With Other Works

Maintaining high-quality health care is of utmost importance. In our study, many providers reported that web-based visits can be a high-quality delivery method for specific appointment types (eg, return visits and medication check-ins) and specific conditions (eg, chronic disease management and mental health needs). For some providers, web-based visits actually improved the quality of care by allowing for more real-world counseling and advice in the patient’s home environment. However, for visits where additional data are required, providers echoed concerns previously raised in the literature that web-based visits may provide a substandard level of care [21-23]. A deeper understanding of what visits are appropriate for telemedicine and better triaging of visit types are crucial steps to ensuring the quality of care is maintained. Similarly, novel approaches to making components of in-person visits available at home through provider training, making home devices available, and expanding options for laboratory testing and imaging may be important methods to improve the availability of objective data for web-based visits [24-27].

Providers have previously expressed concerns about building patient rapport through web-based visits [21]. While some providers in our study echoed these concerns, others noted how web-based visits helped facilitate patient-provider connections through improved patient comfort and the ability to create greater intimacy with a web-based “house call.” Rapport depended on patients having a safe, private space for web-based visits, which was less possible in specialties where the physical examination was central to connection. Incorporating patients’ perspectives will be critical for further understanding relationship-building in the web-based environment and what, if any, changes in health behaviors and outcomes result.

While telemedicine promises to alleviate inefficiencies and inequities by reducing travel and other barriers to in-person care delivery [9,11,12,28], many providers in this study shared how lagging technology, insufficient technical and clinical support, poor connectivity, and digital literacy issues prevent this ideal from being realized. It is clear that to maintain telemedicine services, greater web-based infrastructure is needed, including higher-quality internet connections, more robust training, and ensuring services that in-person staff provide are also accounted for digitally. Similarly, the digital divide threatens to worsen, rather than alleviate, care disparities if efforts are not made to ensure patients at greatest risk of adverse outcomes are not left behind [28].

Providers highlighted several critical local and national policies to maintain telemedicine beyond the acute public health crisis. Some solutions, including more flexible messaging around organizational targets for web-based care adoption and maintaining parity for audio-only and video encounters, highlight the need for tailored care that is responsive to patients’ preferences. Other solutions, including public partnerships to ensure access to broadband, highlight the stark inequities in our current health care system. Several of the policy priorities emphasized in our study have been considered in local and national discussions; however, few protections exist for telemedicine gains made during the public health crisis without major legislative change at the state and federal levels [19,20].

### Limitations

Our study has several limitations. It was conducted at a single institution and may not reflect the perspectives of providers in other geographic locations, serving different patient populations, or in settings with more or less robust telemedicine infrastructure. Additionally, providers who had particularly strong opinions about telemedicine may have been more likely to complete our survey, creating selection bias. Still, we believe that the volume of our qualitative data from diverse experiences provides rich insights to inform the maintenance of telemedicine. Another limitation of our study is that we did not investigate patient perspectives. Future studies should focus on comparing the opinions of both patients and providers to capture the total user experience.

### Conclusions

Web-based visits provide an important opportunity to improve care quality, connection, efficiency, and equity. However, significant challenges threaten to erase gains made in the provision of telemedicine during the public health crisis, particularly for specialties that require some in-person services. Our study highlights providers’ perceptions of the most important local and nationwide efforts needed to maintain web-based visits beyond the COVID-19 pandemic. Through these adaptations, health care can meet patients where they are with high-quality, equitable, and patient-centered services.

## Appendix

Multimedia Appendix 1 Provider survey.

Multimedia Appendix 2 Matrix coding of provider telemedicine experience.

## Abbreviations

HIPAA: Health Insurance Portability and Accountability Act
MA: medical assistant
