# Supplementary material for: Barriers to Telemedicine Use: Qualitative Analysis of Provider Perspectives During the COVID-19 Pandemic
Source: JMIR Hum Factors. 2023 Jun 26;10:e39249. doi: 10.2196/39249 (PMC10337245; doi:10.2196/39249)
Supplement: Multimedia Appendix 1 [file humanfactors_v10i1e39249_app1.docx]

All Departments- Provider Perspectives on Telehealth_active v2

Start of Block: Likert Scale Questions

Q8 This survey has been created to evaluate and help improve the provider experience with Michigan Medicine video visits. When responding, please think to your experience in the last few months and not at the beginning of the pandemic, as we are looking for feedback on the new system (post August 20, 2020). You will be asked for your uniqname, to be used to extract characteristics about the type and volume of video visits you provide. Any data shared outside of the Virtual Care and research team will be blind to uniqnames.  Participating in this survey is voluntary. The time to complete the survey is less than 5 minutes.  
If you have any questions, please contact [TelehealthTeam@med.umich.edu Thank you for your time and engagement.](mailto:TelehealthTeam@med.umich.edu%20Thank%20you%20for%20your%20time%20and%20engagement.)

HUM00193375

| Page Break |  |
| --- | --- |

Q8 Select how much you agree or disagree with the following statement:

|  | Strongly Agree (1) | Moderately Agree (2) | Somewhat Agree (3) | Somewhat Disagree (4) | Moderately Disagree (5) | Strongly Disagree (6) |
| --- | --- | --- | --- | --- | --- | --- |
| I can provide the same quality of care through video visits. (1) |  |  |  |  |  |  |

Q9 (Optional) Additional comments:

________________________________________________________________

________________________________________________________________

________________________________________________________________

________________________________________________________________

________________________________________________________________

| Page Break |  |
| --- | --- |

Q11 Select how much you agree or disagree with the following statement:

|  | Strongly Agree (1) | Moderately Agree (2) | Somewhat Agree (3) | Somewhat Disagree (4) | Moderately Disagree (5) | Strongly Disagree (6) |
| --- | --- | --- | --- | --- | --- | --- |
| I believe I can build the same level of rapport with patients over video as I can in-person. (1) |  |  |  |  |  |  |

Q12 (Optional) Additional Comments:

________________________________________________________________

________________________________________________________________

________________________________________________________________

________________________________________________________________

________________________________________________________________

| Page Break |  |
| --- | --- |

Q13 Select how much you agree or disagree with the following statement:

|  | Strongly Agree (1) | Moderately Agree (2) | Somewhat Agree (3) | Somewhat Disagree (4) | Moderately Disagree (5) | Strongly Disagree (6) |
| --- | --- | --- | --- | --- | --- | --- |
| In general, my patients are able to log on and start the video visit without additional support from me. (1) |  |  |  |  |  |  |

Q14 (Optional) Additional Comments:

________________________________________________________________

________________________________________________________________

________________________________________________________________

________________________________________________________________

________________________________________________________________

| Page Break |  |
| --- | --- |

Q15 Select how much you agree or disagree with the following statement:

|  | Strongly Agree (1) | Moderately Agree (2) | Somewhat Agree (3) | Somewhat Disagree (4) | Moderately Disagree (5) | Strongly Disagree (6) |
| --- | --- | --- | --- | --- | --- | --- |
| Technical issues OFTEN impact my ability to complete video visits after the patient and I connect. (1) |  |  |  |  |  |  |

Q16 (Optional) Additional Comments:

________________________________________________________________

________________________________________________________________

________________________________________________________________

________________________________________________________________

________________________________________________________________

| Page Break |  |
| --- | --- |

Q17 Select how much you agree or disagree with the following statement:

|  | Strongly Agree (1) | Moderately Agree (2) | Somewhat Agree (3) | Somewhat Disagree (4) | Moderately Disagree (5) | Strongly Disagree (6) |
| --- | --- | --- | --- | --- | --- | --- |
| Overall, I am satisfied with doing video visits. (1) |  |  |  |  |  |  |

Q18 (Optional) Additional Comments:

________________________________________________________________

________________________________________________________________

________________________________________________________________

________________________________________________________________

________________________________________________________________

| Page Break |  |
| --- | --- |

Q19 Complete the following statement:

|  | Substantially Fewer (1) | Fewer (2) | The same volume of (4) | More (5) | Substantially More (6) |
| --- | --- | --- | --- | --- | --- |
| After the COVID-19 public health emergency, I plan to offer ______ video visits than I do now. (14) |  |  |  |  |  |

Q33 (Optional) Additional Comments:

________________________________________________________________

________________________________________________________________

________________________________________________________________

________________________________________________________________

________________________________________________________________

Q20 Complete the following statement:

|  | Substantially Lower (1) | Lower (2) | The same (3) | Higher (4) | Substantially Higher (5) |
| --- | --- | --- | --- | --- | --- |
| I feel my productivity is _____ when I conduct video visits as compared to in-person visits. (1) |  |  |  |  |  |

Q34 (Optional) Additional Comments:

________________________________________________________________

________________________________________________________________

________________________________________________________________

________________________________________________________________

________________________________________________________________

End of Block: Likert Scale Questions

Start of Block: Free Response

Q2 What could Michigan Medicine do to help improve the video visit experience?

________________________________________________________________

________________________________________________________________

________________________________________________________________

________________________________________________________________

________________________________________________________________

Q21 Michigan Medicine is committed to providing equitable care for all patients, but we recognize that there may be barriers to video visits in specific populations. Based on your experience, what can Michigan Medicine do to improve equitable access for all patients?

________________________________________________________________

________________________________________________________________

________________________________________________________________

________________________________________________________________

________________________________________________________________

End of Block: Free Response

Start of Block: Demographics

Q3 Please provide your uniqname so we can understand our results in the context of practice variables.


Uniqname:

________________________________________________________________

Q5 What is your role at Michigan Medicine?

- Physician (1)
- Resident or Fellow (7)
- Nurse Practitioner (2)
- Physician’s Assistant (3)
- Registered Dietitian (4)
- Social Worker (5)
- Genetic Counselor (8)
- Other (6) __________________________________________________

| Page Break |  |
| --- | --- |
