# Supplementary material for: Barriers to Telemedicine Use: Qualitative Analysis of Provider Perspectives During the COVID-19 Pandemic
Source: JMIR Hum Factors. 2023 Jun 26;10:e39249. doi: 10.2196/39249 (PMC10337245; doi:10.2196/39249)
Supplement: Multimedia Appendix 2 [file humanfactors_v10i1e39249_app2.docx]

**Multimedia Appendix 2. Matrix coding of provider telemedicine experience**

|  | **Positive/Facilitator** | **Negative/Barrier** |
| --- | --- | --- |
| **Quality of Care** | | |
| **Overall quality** | | |
| Theme | **Some providers noted the quality of care with video visits was the same or better with virtual care.** | **Some providers felt virtual care was an inadequate substitute for in-person care.** |
| Quotes | *When the video and audio quality is adequate, and there is no need for those elements of the exam, the quality of care is also same, if not better, than in person.* (Neurology, Physician) | *Virtual care is an important option to have for certain patients and certain situations. But to pretend that it is even close to equivalent to a real appointment or that it should be the "default mode of care delivery" is just insane and has been one of the most frustrating things.* (Pediatric Gastroenterology, Physician) |
| **Visit type** | | |
| Theme | **Video visits are very effective for some visit types, including medication check-ins, results review, and follow-up visits.** | **Video visits are poor substitutes for in-person care when specific data (e.g., physical exam, tests) cannot be performed.** |
| Quotes | *Imaging follow ups and some post-op visits have been the best use of virtual visits in our practice.*(Orthopedic Surgery, Physician Assistant) | *The lack of ability to perform a physical exam and lack of in person axillary resources (same day laboratory testing, same day radiologic testing, etc.) significantly limits the ability to provide quality of care equivalent to in person visits.* (Internal Medicine, Resident/Fellow) |
|  | *If the primary goal is symptom management and review of labs and radiology, virtual care works really well.* (Internal Medicine, Physician) | *Sometimes patients do not have access to a blood pressure machine or other tools to measure their vitals, which would be available in the clinic and are needed to provide a high quality visit.* (Internal Medicine, Physician) |
| Theme | **Some diagnoses and specialties were seen as particularly well suited for video visits.** | **Even when providers attempt an exam in the virtual environment, it is low quality.** |
| Quotes |  | *Physical exam is challenging as pts are often in lighting that makes the exam difficult or their camera quality through their internet provider is poor.* (Plastic Surgery, Physician Assistant) |
|  | *For diabetes and htn with accurate home monitoring and anxiety /depression managemt video is adequate and better in some ways, as it is easier for the patient to keep the MD visit.* (Internal Medicine, Physician) |  |
| Theme | **For some visits, seeing a patient in their home provided important data points that improved overall visit quality.** | **Video visits make it difficult to complete preventive care services, such as vaccinations or monitoring growth.** |
| Quotes | *Seeing patients in their home environment provides a better exam than we get in clinic for some things.* (ENT, Physician) | *If a child is behind on an immunization, I cannot take care of that at a visit for another issue- which makes it difficult to improve immunization rates.* (General Pediatrics, Physician) |
| Theme |  | **Scheduling of video visit for certain diagnoses when the provider felt in-person care was required led to frustration for providers and patients.** |
| Quotes |  | *I have had visits scheduled that did not have a clear "chief complaint" which resulted in very unproductive visits when it became clear a physical exam was necessary.* (Obstetrics and Gynecology Resident/Fellow) |
| Theme |  | **Providers cited greater difficulty coordinating services for their patients.** |
| Quotes |  | *The video visits are generally shorter than in-person appointments, but I spend more time on the back end trying to coordinate labs, imaging, follow up etc. compared to an in person visit. (Neurology, Physician)* |
| **Patient population** | | |
| Theme | **Video visits are a good alternative for some patient populations who have higher barriers to in-person care, like those who are sick, face disabilities, or have transportation barriers.** | **Video visits are not a good solution for many patient populations.** |
| Quotes |  | *Very difficult to get information from child via VV when one or both parents tend to dominate the screen time.* (Gastroenterology and Hepatology, Physician) |
|  | *There are rare exceptions but the gains associated with video visits (including greater access to care for patients with neurological disabilities affecting their mobility, ability to integrate family members who live far away into the discussion, etc.) more than make up for this.* (Neurology, Physician) | *I am a medical oncologist… the vast majority of my patients are sick, on intensive chemo, receiving bad news, and dealing with end-of-life issues. In these cases, the quality of care is definitely lower via video visit than it is in person.* (Hematology and Oncology, Physician) |
| **Counseling/ education** | | |
| Theme | **Video visits allow more time for focused counseling.** | **The virtual environment can make counseling more challenging without visual aids or hands on assistance with devices.** |
| Quotes | *I have been successful in counselling, and when I see the patients back in office, am pleasantly surprised with improvements reported by changes made with behavioral modifications discussed during initial video visit.* (Urology, Physician Assistant) | *Patients are not present to get inhaler teaching if needed (learning how to use an inhaler can be complicated).* (Pulmonary and Critical Care, Physician) |
| Theme |  | **Providers reported challenges providing high quality care due to patient distractions.** |
| Quotes | *.* | *Sometimes families seem more distracted during video visits than they would be in the controlled environment of a clinic and I worry that pieces of information may be getting lost.* (Other, Physician) |
| **Patient Rapport** | | |
| **Establishing rapport** | | |
| Theme | **Some providers noted an overall positive experience establishing rapport with patients.** | **Other providers found it difficult to establish rapport through video visits.** |
| Quotes | *Most of the video visits have gone well and I am able to establish the same rapport with the patient.* (Other, Physician) | *But there are a good number of patients who I feel [establishing rapport] is not possible, both for technological and cultural reasons.* (Infectious Diseases, Physician) |
|  | *I feel a stronger connection with patients that I have been able to have video visits with.* (Hematology and Oncology, RN) | *Developing rapport over video visits is much more difficult. Losing the in person interaction makes communication more difficult, especially for new patients with sensitive or challenging medical problems.* (Pediatric Hematology and Oncology, Physician) |
|  |  | *There is not the same level of trust and connection in the virtual format.* (Pediatric Hematology and Oncology, Physician) |
| **Environmental** | | |
| Theme | **Video visits allow for a virtual “home visit” facilitating greater intimacy in the patient provider relationship.** | **Some patients are embarrassed by their home environment, making it difficult to establish a relationship.** |
| Quotes | *For virtual appropriate things, YES, same quality (and sometimes better - I can see people in their homes/places of work - new kind of intimacy, kind of like a modern day house call - I love it and the patients do too)!* (Obstetrics and Gynecology, Physician) | *Many people struggle with clutter and are embarrassed to have people in the home. They often don’t admit that, but, will decline home nurse or PT* (Internal Medicine, Nurse Practitioner) |
|  |  | *Distributing or standardizing backgrounds for both providers and patients to make sure that families are not discouraged by someone seeing their living situation.* (Pediatric Nephrology, Physician) |
| Theme | **Some patients are more comfortable at home than in the office.** | **Some patients do not have a private space to conduct their video visit, making connections difficult.** |
| Quotes | *Actually better rapport with kids, because they are in their home environment and comfortable, rather than being in an office setting as a "patient." They show me their dog, their favorite book, etc. They seem very comfortable and less nervous.* (Pediatrics, Physician) |  |
|  |  | *Half of the patients are clearly doing something else while talking to me, the other half are hiding in a closet or bathroom bc they don't have privacy. Those patients are NOT getting the same quality of conversation they would have in my office… I can't establish the same rapport. It's disheartening for me as a provider and I dislike it for them.* (General Surgery, Physician) |
|  |  | *Patients need education on how video visits are the same as being at the doctor. They can't be driving, at the check-out at Kroger, using the toilet (!!!), smoking weed or washing dishes.* (PM&R, Physician) |
|  |  | *Some of my patients don't have a safe place to do these visits (one of my patients did his in his car because he didn't feel comfortable doing it in his home).* (Hematology and Oncology, Physician) |
| **Physical** | | |
| Theme | **Video visits allow providers to see their patients’ faces during the COVID-19 pandemic when masking is required in-person.** | **The “laying on of hands” cannot be used as a method of connecting with patients virtually.** |
| Quotes |  | *Easier to establish connection with people in person and by doing an exam.* (Urology, Physician) |
|  | *Can see their face since not needing to wear a mask. Helps for facial expressions, especially smiles.* (Urology, Physician) | *I am in a specialty that requires more hands-on physical exam. That helps build trust and rapport that is impossible over video.* (Orthopedic Surgery, Physician) |
| Theme |  | **Some providers reported they were better able to connect with patients in-person.** |
| Quotes |  | *I also think face to face visits for new patients is optimal for establishing a good patient-physician relationship.* (Cardiovascular Medicine, Physician) |
|  |  | *Pts need to have face-to-face interactions with their provider. A computer screen or telephone encounter does not provide the complete pt picture & it is a complete disadvantage to the pt.* (Internal Medicine, Nurse Practitioner) |
| **Visit Flow** | | |
| **Efficiency** | | |
| Theme | **Some providers noticed reduced no-show and cancellation rates, allowing them to more effectively use blocks of clinical time.** | **Some providers noted higher no-show and cancellation rates.** |
| Quotes | *I think it has decreased our no show rate & helped us reach patients who are farther away with more frequency.* (Pediatric Hematology and Oncology, Nurse Practitioner) | *My perception is that the no-show rate is higher for video visits than it is for in-person visits. I don't know if this is because of scheduling issues (i.e., patients not being informed that they have an appointment), patients feeling like it is easier to just skip a video visit and not call to let us know, or some other reason.* (Hematology and Oncology, Physician) |
|  | *I work with a very functionally disabled population, and video visits help to lower no-show rates and the expense of transportation by ambulance or wheelchair van.* (Internal Medicine, Physician) | *Patients need a separate phone call to help them with the software interface, or fail and no-show for an appointment.* (Neurosurgery, Physician) |
|  | *More patients with lower income show up.* (Psychiatry, Physician) | *Some patients do not interact with the same level of respect during a video visit as in the office. It is like the doctor is intruding on their time and they ask the doctor to call back when it is more convenient for them.* (Internal Medicine, Physician) |
|  | *Monday morning is always difficult as patients wake up late, look at the weather and call in to change or cancel appt... Now they call in and want a video visit rather than in person or just don't show up. the attrition from video visit is WAY less for me.* (Hematology and Oncology, Physician) |  |
|  | *Extended time on video visits is draining in a way that in-person visits are not. This is exacerbated because of a lower no-show rate. Building in 15-minute breaks to schedules would help a lot with this.* (Psychiatry, Physician) |  |
| Theme | **Some providers noted they saved time without the traditional rooming processes, and could immediately start seeing their patients.** | **Insufficient patient and provider training meant visits were often delayed due to user issues connecting and using video visit software.** |
| Quotes | *No waiting in waiting room; no delay for MA, easy no rush once on the call, labs and other testing can be obtained and ready for the visit.* (Cardiovascular Medicine, Physician) | *Patients are having trouble logging in and it ends up as a phone visit*. (Cardiovascular Medicine, Physician) |
|  |  | *The signon procedures are too complicated for some (older and less tech savvy) folks.* (Nephrology, Physician) |
|  |  | *There are too many issues prior to starting the video that can become very time consuming.* (Neurology, Resident/Fellow) |
|  |  | *Time is lost as patients don’t know how to turn on the sound.* (Rheumatology, Physician) |
|  |  | *SO much service recovery it's time consuming, unhelpful and not about patient care.* (Orthopedic Surgery, Physician Assistant) |
| Theme |  | **Interspersing video and in-person visits in clinic sessions was not effective for providers.** |
| Quotes |  | *When virtual visits are mixed in between in person visits, virtual visits tend to be more delayed.* (General Medicine, Physician) |
|  |  | *I have f/f [face to face], phone, and video visits all in the same clinic. Very hectic when you lose time with trying to get people connected.* (Cardiovascular Medicine, Nurse Practitioner) |
| **Visit support** | | |
| Theme |  | **Without consistent support from medical assistants and clerical staff, medication reviews, questionnaires, and check-out procedures that are typically completed in-person were often missed.** |
| Quotes |  | *I can only do video if I get better staff support (MA, rooming, in-visit questionnaire completion) and post visit check out.* (Family Medicine, Physician) |
|  |  | *There is dire need for support to populate the past medical/surgical/social/family/meds/allergies/ROS prior to the visit. If I have 20 minutes to do a new patient visit, I can't spend 15 minutes filling in the background; I need to be able to focus on the problem and medical decision making. This lack of support for video visits is a huge concern.* (Urology, Physician) |
| Theme | **Institutional resources improved patients’ ability to connect to video visits.** | **Providers noted a need for improved technology support to address issues in real time.** |
| Quotes | *The med student led initiative GetAcces has been very helpful. MiChart interface is not easy to set up for most people.* (Psychiatry, Physician) | *I think someone needs to contact the patient and help them get on the meeting so when I logon they are on the meeting. I spend over half of my visit trying to help them logon and have very little time to actually address the problem or do my documentation.* (Orthopedic Surgery, Physician Assistant) |
|  |  | *Provide patients with access to rapid at the elbow support when they are struggling at the beginning of a visit. I have had to walk patients through when they are struggling and it can take up 10mins of my 20min return visit.* (Internal Medicine, Physician) |
|  |  | *As we have interpreters for patients with language barriers, would there ever be a "tech-barrier interpreter". You never know unless you ask.* (Urology, Physician Assistant) |
| **Documentation** | | |
| Theme | **Some providers noted lessened documentation burden because they could more easily complete charting during the video visit.** | **Providers reported increased documentation burden for fields that otherwise would have been completed by other staff.** |
| Quotes | *Because on video I can type AND make eye contact at the same time, I'm often able to start and complete my patient exam note while I'm still in the patient visit. This is a HUGE satisfier for me. Really, I cannot overstate how much this makes me love my job more. I would actually consider increasing my cFTE if I could do predominantly video visits.* (Internal Medicine, Physician) | *Charting is more complete with in person visits. More loose ends in care that need to be tied up at end of session with virtual visits.* (General Medicine, Physician) |
|  | *No physical exam, so the appointments are shorter obviously. for patients who don't need an exam, it's nice, becusae I use that time to document and complete the note. I find that I complete virtual visit notes on the same day way more often than in person visits.* (Radiology Oncology, Physician) | *Me having to often do med, allergy, and tobacco documentation takes time, finding their questionnaires is not always easy.* (Family Medicine, Physician) |
|  | *It is easier to document while I am doing the visit on video visits. In person, computers are often in the corner so you cannot document and look at the patient and it can take a while to log on.* (Internal Medicine, Resident/Fellow) | *The time required to see/document a visit by video versus in-person when have clerk and MA support is substantially more.* (Internal Medicine, Physician) |
|  |  | *Patients are scheduled back to back without any spare time to document. At least in clinic you have a bit of time while the patient is roomed.* (Obstetrics and Gynecology, Physician) |
|  |  | *Difficult to make high quality rapport with patient and document at same time, thus will try to document after encounter that will usually have to wait until end of day.* (Dermatology, Physician Assistant) |
|  |  | *The current setup with seeing video visits myself, outside of clinic time, rather than during established clinic time with a fellow, does mean more time for interviewing and MiChart.* (Pediatric Cardiology, Physician) |
|  |  | **Some providers noted an increased volume of in-basket (electronic health record) messages from patients.** |
|  |  | *Virtual care seems to cause a great deal more in-basket messages and thus busy work between visits.* (Internal Medicine, Physician) |
| **Patient and provider experience** | | |
| Theme | **Providers noted improved experience for patients, who had reduced travel time and could do other activities while waiting for their visit.** | **Technical difficulties, including issues with software, broadband, and need to convert to video visits, made for frustrating experiences for patients and providers.** |
| Quotes | *If it is a counseling or other appointment that an exam is not required, I would argue that the care is exactly the same if not better as patient's don't have the added stress of travel/parking/checking in/out.* (Obstetrics and Gynecology, Physician) | *There are still frequent technical hurdles which are frustrating to patient and providers.* (Hematology and Oncology, Physician) |
|  | *[I am less productive] Because of the extra work I have to do. That being said it is SO MUCH MORE RESPECTFUL to my patient's time and I'm happy to make video visits available to reduce disparities for people who truly can't come into the office.* (Family Medicine, Physician) |  |
| Theme | **Some providers noted how video visits improved their personal quality of life and job satisfaction.** | **Some patients completing video visits seemed to miss the human contact from ancillary staff members:** |
| Quotes |  | *I don’t have to wait on the MAs to room patients which keeps me more likely to be on time, however parents seem lonely and want to talk longer than their visit time so it seems to overall make me run slightly late.* (Other, Physician) |
|  | *I can stay on time when I conduct video visits, but I always run behind in my FTF clinics (patients are late, MAs take too much time to room patients, etc). This leads to substantially higher satisfaction for patients and myself and allows me to also have more time to attend to my inbasket. AND means I can predictably know when my day will end, which improves my home life as well.* (Gastroenterology and Hepatology, Physician Assistant) |  |
| **Equity** | | |
| **Access** | | |
| Theme | **Some providers saw video visits as a way to reduce care disparities for populations with barriers to care like rural and homebound patients.** | **Many providers worried video visits could further exacerbate existing inequities for patients without access to devices or broadband.** |
| Quotes | *Benefits of improved access, happy patients (I see many patients with significant disability, they are thrilled to not have to leave home, especially in Covid, and especially in the winter when wheelchairs are a bigger challenge).* (PM&R, Physician) |  |
|  | *It has a HUGE advantage for access, allowing patients to be seen if they live further away, and not forcing patients to take a half day off of work for a doctor's appointment.* (Psychiatry, Physician) | *I do worry a lot about my patients who have limited technology access if we try to push people to this modality.* (Neurosurgery, Physician) |
|  | *One of my patients often will have a seizure when going out in public due to agitation/lights/car ride. His parents loved the VV as this was a safe and effective option for him.* (Gastroenterology and Hepatology, Physician) |  |
| Theme | **Phone visits were seen as an important tool for ensuring access for patients with barriers to telemedicine.** |  |
| Quotes | *I think remote visits have increased access to care for people who usually have difficulties coming to the clinic, so if setting up video visits is impossible, I think patients should receive the service at least by phone.* (Neurology, Resident/Fellow) |  |
|  | *I am thankful for telephone visits being reimbursable and hope that continues for these patients. Valuable service especially when there are truly no other options.* (Pulmonary and Critical Care, Nurse Practitioner) |  |
|  | *Also need means to afford data costs with video, so a telephone option must continue to not discriminate based on disabilities, age or financial means.* (Psychiatry, Physician) |  |
|  | *I also find phone visits necessary for a large number of elderly and patients who are younger as well, wish these were given equal credit as an incentive measure.* (Family Medicine, Physician) |  |
| **Trust** | | |
| Theme |  | **Some providers noted that patient populations with a history of poor treatment by the medical system may have greater concerns about video visits, including fears of being recorded or receiving substandard care.** |
| Quotes |  | *In addition to some patients having lack of needed equipment, there is also some mistrust of VV in our at risk populations. I have had some tell me they don't want the VV option, just phone as they are concerned about being recorded.* (General Medicine, Physician) |
|  |  | *Some see it (maybe correctly) as continued monetization of the doctor patient relationship. Billing every interaction creates a bit of a wall - moving toward a better payment model in the future might help fix this as well.* (General Medicine, Physician) |
|  |  | *This technology is eroding the doctor-patient relationship significantly.* (Plastic Surgery, Physician) |
